# Migration of immortalized nasopharyngeal epithelia and carcinoma cells through porous membrane in 3D platforms

**DOI:** 10.1042/BSR20194113

**Published:** 2020-06-04

**Authors:** Ziyu Liu, Weiguan Zhang, Stella W. Pang

**Affiliations:** Department of Electrical Engineering, Centre for Biosystems, Neuroscience, and Nanotechnology, City University of Hong Kong, Hong Kong

**Keywords:** 3D biomimetic platform, cell migration, nasopharyngeal carcinoma (NPC), traversing through porous membrane

## Abstract

In the present study, 3D biomimetic platforms were fabricated with guiding grating to mimic extracellular matrix topography, porous membrane to resemble the epithelial porous interface and trenches below to represent blood vessels as an *in vitro* tissue microenvironment. Fabrication technologies were developed to integrate the transparent biocompatible polydimethylsiloxane platforms with preciously controlled dimensions. Cell migration behaviors of an immortalized nasopharyngeal epithelial cell line (NP460) and a nasopharyngeal carcinoma cell line (NPC43) were studied on the 2D and 3D platforms. The NP460 and NPC43 cells traversing through the porous membrane and migrating in the trenches below were studied by time-lapse imaging. Before traversing through the pores, NP460 and NPC43 cells migrated around the pores but NPC43 cells had a lower migration speed with less lamellipodia spreading. After traversing to trenches below, NPC43 cells moved faster with an alternated elongated morphology (mesenchymal migration mode) and round morphology (amoeboid migration mode) compared with only mesenchymal migration mode for NP460 cells. The cell traversing probability through porous membrane on platforms with 30 μm wide trenches below was found to be the highest when the guiding grating was perpendicular to the trenches below and the lowest when the guiding grating was parallel to the trenches below. The present study shows important information on cell migration in complex 3D microenvironment with various dimensions and could provide insight for pathology and treatment of nasopharyngeal carcinoma.

## Introduction

Nasopharyngeal carcinoma (NPC) is found in the nasopharynx, the upper most region of the throat. It is often diagnosed at late stages [1–4]. NPC is uncommon in most countries but there is a much higher frequency of occurrence in Alaska and southern China [4–7]. NPC is also one of the rare Epstein–Barr virus (EBV) positive cancers [8] despite the virus does not cause major symptoms in the majority of lifelong carriers. Different NPC cell lines were established but it is difficult to keep the EBV in the cells [8–11]. In order to have a better understanding of the pathological and diagnostic process, it is important to study the NPC metastatic dissemination process, which includes cell migration, invasion, circulation, intravasation, extravasation, and metastasis [12–15]. Proper animal models for NPC are still under investigation [8–11], and they are costly and associated with ethical concerns. Therefore, efforts have been made to develop biomimetic models [16,17]. Engineered platforms mimicking the microenvironment of NPC for studying NPC cell migration and invasion will provide a better understanding of NPC metastatic process.

Previous studies mostly focus on many cell interactions on 2D dishes or in 3D gels as it is much more difficult to fabricate 3D biomimetic engineered models [18–23]. However, 2D models do not include cell interactions in the vertical direction and are very different from the real microenvironment. Thus, it is critical to establish 3D models, which can better represent the extracellular matrix (ECM) *in vivo* and to understand circulating tumor cells in the vascular system. Most 3D models till now are related to gel, porous plates, or microfluidic chips to study cancer responses [17,24–26]. Often the channel size is as large as several millimeters due to the fabrication process [17,26], and it is different from some of the blood vessel size [27,28]. Other 3D models made from hydrogels [29,30], membrane-based polydimethylsiloxane (PDMS) micro-bioreactor [31] and microvascular-based channels [32] have been reported. Most of these platforms did not have precisely controlled channel/pore size, or they did not provide structures to mimic ECM and blood vessels.

Although fibroblast and cancer cell migration on 2D platforms with grating, arc, and angular grating guiding patterns have been studied [22,23,33], the cell migration behavior for cell interaction of nasopharyngeal carcinoma on 3D platform remains unclear. In the present study, a three-layer biomimetic model was designed and fabricated to mimic the ECM topography, the epithelial porous interface, and the underlying blood vessels in a typical tissue. Various fabrication technologies including replication from mold, double-sided imprint, and plasma bonding in transparent biocompatible PDMS were developed to integrate multiple layers in 3D platforms with preciously controlled channel and pores dimensions. An immortalized nasopharyngeal epithelial cell line (NP460) and a nasopharyngeal carcinoma cell line (EBV positive NPC43) were seeded on the 2D and 3D platforms, and time-lapse images were used to study cell migration and motility. By visualizing NP460 and NPC43 cells traversing through the porous membrane and migration in the trenches below, the cell migration behaviors for these two kinds of cells were investigated. The traversing behaviors of NP460 and NPC43 cells were found to be controlled by the guiding grating orientation on top and the trench size below. Our previous study [33] shows that platforms with patterned topography could reveal metastasis of human cancer cells. Cells showed different migration speed and directionality when they came from different histological origins. In addition, on platforms with various topographies, cells from the same origin but different cancer subtypes showed distinctive behavior. It is expected the same principle can be applied to different types of cancer cells with properly designed platforms.

## Materials and methods

### Fabrication technology for 3D biomimetic platform

**Figure 1:**
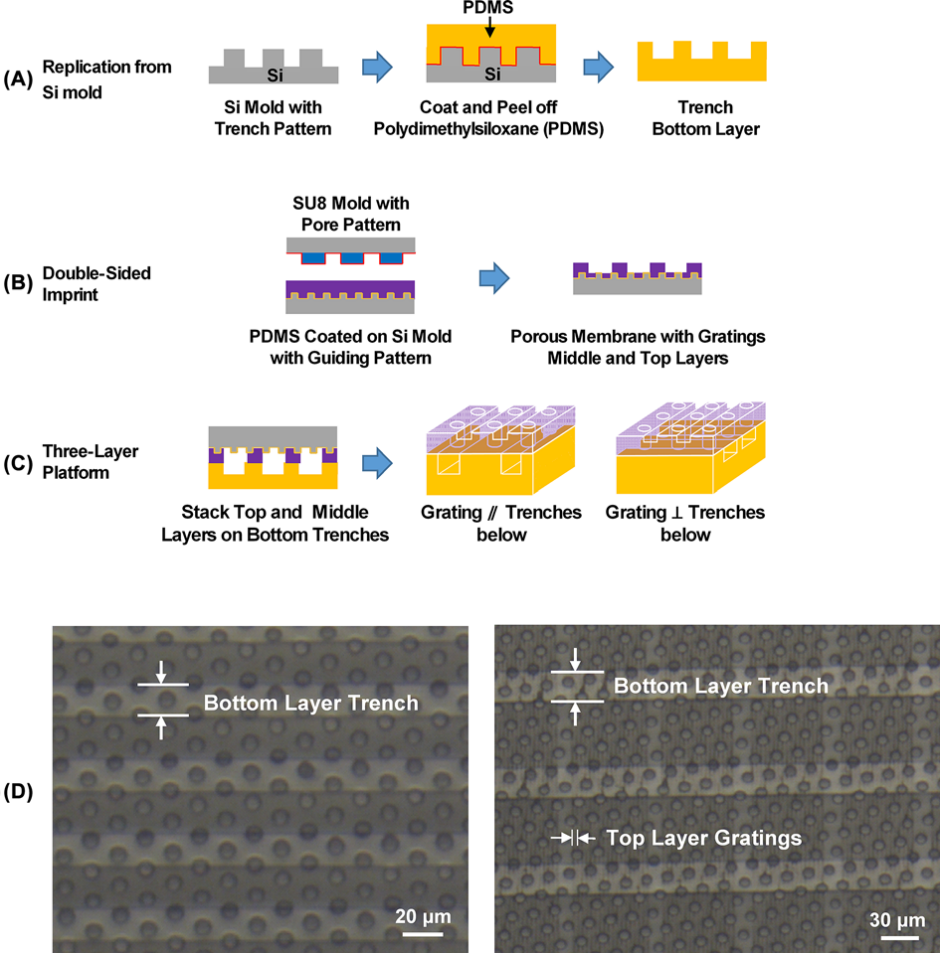
Fabrication of 3D biomimetic platform. (**A**) Bottom layer with trenches obtained by demolding polydimethylsiloxane (PDMS) from Si mold. (**B**) Middle and top layers fabricated by using SU8 mold with pores and Si mold with guiding grating. (**C**) Stacking top and middle layers on bottom trenches, and 3D drawings with gratings parallel/perpendicular to trenches. (**D**) Micrographs of 3D platforms with 2 μm wide and 1 μm deep guiding grating on top layer, 10 μm diameter and 14 μm thick pores in middle layer, and 20–30 μm wide, 15 μm deep trenches.

One-, two-, and three-layer platforms were designed and fabricated with a biocompatible transparent PDMS. As shown in Figure 1A, one-layer substrates with gratings or pores were formed by a molding technique as previously reported [23]. A Si mold patterned by photolithography and deep reactive ion etching (DRIE) was 15-µm thick, and it was coated with an anti-sticking layer, trichloro(1H, 1H, 2H, 2H-perfluorooctyl)silane (FOTS) at 80°C for 2 h. A PDMS (Dow Corning Sylgard 184 kit) mixture including pre-polymer and curing agent with a mass ratio of 10:1 was poured on the patterned Si mold and degassed in a vacuum chamber. The mixture was baked at 80°C for 8 h on a hotplate. The bottom layer with trenches was formed by peeling the PDMS layer from the Si mold.

Two- and three-layer platforms were both formed by the three steps as shown in Figure 1A–C. The two-layer platform was formed by attaching the porous membrane to a flat substrate while the three-layer platform was formed by attaching the porous membrane to a patterned substrate with trenches as shown in Figure 1C. The porous membrane layer with guiding grating on top was formed simultaneously by the double-sided imprinting of the PDMS layer using a SU-8 mold with patterned pores and a Si mold with patterned grating as shown in Figure 1B. The pore-patterned mold with pores was fabricated in 14 µm thick SU-8 photoresist as shown in Figure 1B, while the grating-patterned Si mold was made by UV-patterning of SPR6112 positive resist and DRIE.

In order to peel off the imprinted PDMS structure, the SU-8 mold with pores and the Si mold with gratings were coated separately with FOTS (surface energy was 71 ± 3 mN/m) and a lower surface energy anti-stacking layer of 4:1 3-methacryloxypropyltrichlorosilane:FOTS (surface energy was 23 ± 3 mN/m) at 80°C for 2 h. To avoid any residual layer on the porous membrane, reactive ion etching (RIE) was performed on imprinted structures with 5 sccm O_2_ and 20 sccm SF6 at 10 mTorr and 250 W RF power for 5 min before bonding to the bottom layers as shown in Figure 1B. To attach the porous membrane layer to a flat substrate or the bottom layer with trenches as shown in Figure 1A, both layers were treated with an O_2_ plasma for 1 min with 20 sccm O_2_ at 80 mTorr and 60 W RF power. The two layers were attached together right after the plasma treatment and baked at 80°C for 10 min on a hotplate. As shown in Figure 1C, the porous membrane was then bonded to the bottom layer with trenches and demolded from the Si substrate to form a multilayer-platform. Figure 1C also shows the 3D drawings of the multiple-layer platforms with gratings parallel or perpendicular to the trenches in the bottom layer. Figure 1D shows the top view of a two-layer and a three-layer platforms. The dimensions of the trenches, pores, and gratings for the 3D platforms could be precisely controlled to investigate cell traversing behaviors in different confined environment systematically.

### Cell culture and assays

Immortalized nasopharyngeal epithelial NP460 cells and nasopharyngeal carcinoma NPC43 cells infected with EBV were used in the present study. The NP460 cells were maintained in the medium containing 1:1 mixture of EpiLife® medium (Gibco) with 1% EpiLife® defined growth supplement (EDGS, Gibco) and defined Keratinocyte-SFM (1×, Gibco) medium, 0.2% defined Keratinocyte growth supplement (Gibco), and 1% antibiotic antimycotic (Gibco; 100 units/ml penicillin G sodium, 100 µg/ml of streptomycin, and 0.25 µg/ml of amphotericin B). The NPC43 cells were cultured in the medium using the Rosewell Park Memorial Institute 1640 medium (Gibco), supplemented with 10% fetal bovine serum (FBS, Gibco), and 1% antibiotic antimycotic (Gibco; 100 units/ml penicillin G sodium, 100 µg/ml of streptomycin, and 0.25 µg/ml of amphotericin B). The medium was changed every 2 days and an incubator was used to keep the cells at 37°C in 5% CO_2_.

### Time-lapse imaging

The one-, two-, and three-layer platforms were placed separately onto 35 mm glass bottom confocal dishes (SPL Life Sciences). O_2_ plasma treatment for 1 min with a flow rate of 20 sccm O_2_ at 80 mTorr and 60 W RF power was performed to increase hydrophilicity of these platforms needed for cell attachment. Typically, NP460 and NPC43 cells were seeded at a density of 8 × 10^4^ cells/ml on one-layer PDMS substrates and at a density of 2 × 10^5^ cells/ml on two- and three-layer platforms. The NP460 and NPC43 cells were incubated at 37°C in 5% CO_2_ in a humidified incubator for 6 and 5 h, respectively, after seeding. The cell density was chosen so that there would be enough cells for over 15 h cell migration tracking but not too many to limit cells interacting with one another to avoid the complications induced by cell interactions.

After the initial attachment of these two cell lines on the platforms, the medium was replaced by the 1:1 mixture of NP460 or NPC43 culturing medium and CO_2_-independent medium. CO_2_-independent medium was prepared by mixing pure CO_2_-independent medium (Invitrogen 18045-088) with 10% FBS, antibiotic–antimycotic (100 units/ml of penicillin, 100 µg/ml of streptomycin, and 0.25 µg/ml of amphotericin B) and 2 mM alanyl-L-glutamine. A scanning confocal inverted microscope (Leica TCS SP5) and a scanning Nikon Eclipse Ni upright microscope were used to image cells kept in incubation chambers at 37°C. Images were captured every 5 min over a period of 15 h to record the cell trajectories and morphologies simultaneously.

### Data analysis

In this work, individual cells that did not divide or physically interact with other cells were analyzed using the NIH ImageJ (version 1.48) software with manual tracking and image stabilizer plugins. Cell speed for one-, two-, and three-layer platforms were calculated and analyzed by Microsoft excel and Origin 8.0. Comparison of cell migration speed along the top grating orientation and perpendicular to the grating orientation was made. Some cells moved around the pores to sense the pores, trenches below, and their sidewalls with some traversing through the pores while some moving away from the pores. To identify the traversing time, the time interval between the cell seeding time and the time when the cells started to squeeze in the pores to traverse through the porous membrane was calculated. Cell traversing probability was defined as the number of cells traversing to the bottom layer during the 15 h imaging period divided by the total number of cells on the top layer when imaging was stated. An optical microscope and a scanning electron microscope (SEM) were used to study the cell morphology. An one-way analysis of variance (ANOVA) with Dunnett’s post-hoc analysis was used to analyze the statistical difference between groups. All data were obtained from at least three runs and the results were shown as mean ± standard error of the mean.

### Scanning electron microscopy

To prepare the cells for high-resolution imaging, they were cultured on the PDMS platforms for 20 h. Both NP460 and NPC43 cells were rinsed with 1% phosphate-buffered saline in a 37°C water bath, followed by fixation with 4% paraformaldehyde for 30 min. These fixed cells needed to be dehydrated through a series of increasing ethanol concentration (30%, 50%, 70%, 80%, 90%, 95%, and 100%). A critical point drying machine was used to complete the dehydration process with CO_2_ as the transitional medium. The cells were imaged using a SEM (Hitachi, SU5000) after they were coated with a thin Au film by evaporation.

## Results and discussion

### 3D biomimetic platform

**Figure 2:**
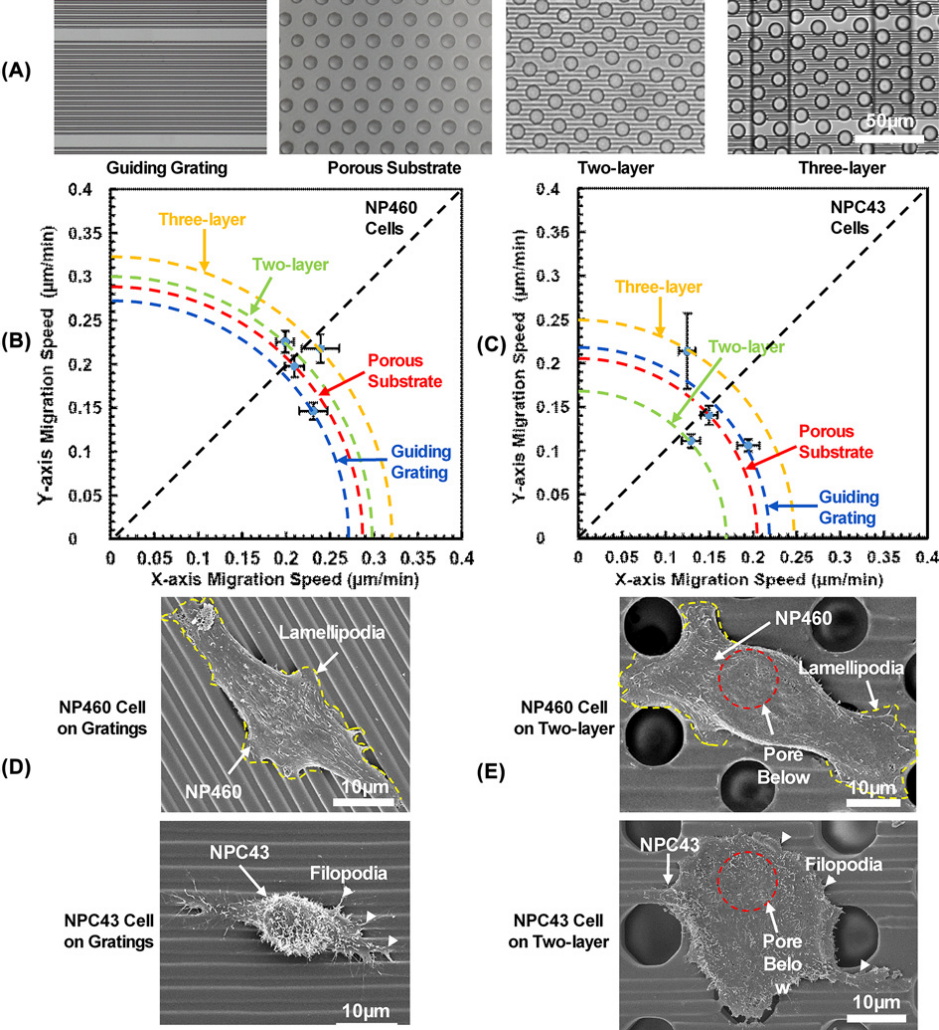
Migration directionality and cell morphology on various platforms (**A**) Micrographs of guiding grating, porous substrate, two-, and three-layer platforms. Cell migration speed of (**B**) NP460 and (**C**) NPC43 cells along and cross guiding grating orientation on grating substrate, porous substrate, two-layer platform with guiding grating and pores, and three-layer platforms with guiding grating, porous membrane and trenches below. Guiding grating orientation was along *x* direction and trench orientation below was along *y* direction (*N*>30). NP460 and NPC43 cell morphology on (**D**) grating substrate and (**E**) two-layer platform. Yellow line indicates lamellipodia of NP460 cells and white arrows point out filopodia of NPC43 cells. Red line represents pore outline.

Although cell migration on 3D microenvironment has been studied [29–31], there were few 3D platforms with precisely controlled dimensions and cell placement that mimicked the microenvironment of a tissue with an ECM topography, an epithelial porous interface, and an underlying blood vessels. To investigate how the 3D biomimetic microenvironment could influence the cell migration speed, directionality, and traversing probability through a porous membrane, engineered 3D platforms with precisely controlled dimensions and surface topographies were designed and fabricated. Figure 1A–C shows the design of the biomimetic platform. The three-layer platform consisted of guiding gratings mimicking the ECM topography, porous membrane resembling the epithelial porous interface, and the trenches below representing blood vessels in typical tissues with cells and blood vessels. Figure 1D show the fabricated three-layer platform from top and side view. Guiding gratings on the top layer were 2/2 μm wide ridge/trench and 1 µm deep. Pores in the middle layer were 10 μm in diameter and 14 µm deep. The total thickness of the guiding grating and porous membrane was 15 μm, which is close to the thickness of epithelia cells around blood vessels. Ridges and trenches on the bottom layer were 40/10, 30/20, 70/30, and 50/50 μm wide and 15 µm deep, which mimicked different topography and blood vessel size *in vivo*. In order to reveal the effects of the guiding grating, porous substrate, and combined layers on the cell migration behaviors, one-layer platforms including grating substrates with 2/2 μm wide ridge/trench and 1 µm deep, porous substrates with 10 μm diameter and 15 µm deep, and two-layer platforms with guiding gratings and porous substrates as shown in Figure 2 were also designed and constructed.

### Cell directionality of NP460 and NPC43 cells on one-, two-, and three-layer platforms

Figure 2B,C show the migration speed of NP460 and NPC43 cells on different platforms calculated from the time-lapse images taken with the guiding grating orientation aligned along the *x*-axis as shown in Figure 2A. On platforms with only guiding grating, cell migration speed of these two types of cells along the grating orientation was higher than the speed perpendicular to the grating orientation, which indicated that cells preferred to migrate along the guiding grating orientation. Figure 2D shows the morphologies of NP460 and NPC43 cells on top of the gratings. The cells elongated along the gratings and this guidance effect was consistent with previous results that MC3T3 fibroblast cells elongated and followed the grating orientation [23]. On porous substrates, NP460 and NPC43 cells showed no orientation preference. NP460 cells mostly migrated around the pores while NPC43 cells mostly confined inside the pores as shown in Supplementary Figure S1. The cell migration trajectories were obtained from more than three runs. This may be due to the shape and morphology differences between the NP460 and NPC43 cells. On the two-layer platforms with grating and pores but no trenches below, NP460 and NPC43 cells showed no preferred migration direction either along or across the gratings. This indicated that the guidance effect of the gratings was inhibited as the cells spread on top of the pores on the two-layer platforms as shown in Figure 2E.

**Figure 3:**
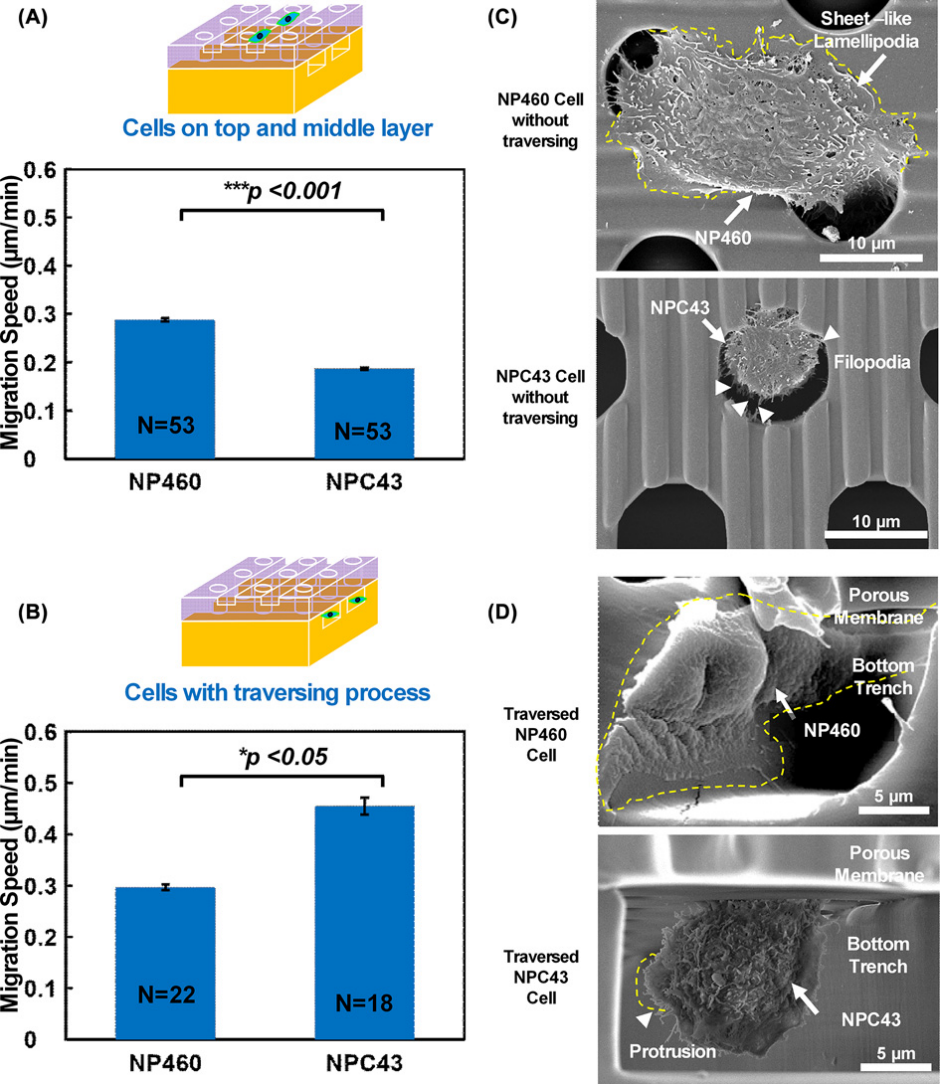
Migration speed and cell spreading on different layers Cell migration speed on three-layer platform (**A**) for cells migrating only on top and middle layer, and (**B**) for cells traversing through porous membrane to bottom layer. NP460 and NPC43 cell morphology (**C**) on top and middle layer without traversing process and (**D**) after traversing. Yellow line indicates lamellipodia and red line represents pore outline. *P<0.05 for cells with traversing process and ***P<0.001 for cells on top and middle layer.

On three-layer platforms with the trenches below, both types of cells could traverse through the porous membrane with the migration speed shown in Figure 3. As shown in Figure 2B, with the grating orientation perpendicular to the trench orientation, NP460 cells expressed no migration direction preference, while NPC43 cells tended to migrate along the trench orientation. The cell migration directionality could be expressed by deviation angle, which is the angle between grating orientation and the average cell migration direction, with 45° representing random movement with no guidance. The deviation angle was 43° for NP460 cells with little guidance, while NPC43 cells had deviation angle of 30°, indicating a better guidance along the grating orientation. Most of the NP460 cells spent time to move around the pores that resulted in NP460 cells taking longer time to traverse through the pores, instead of being guided and migrated along the trenches. The limited movement around the pores was also related to the lower NP460 cell migration speed. On the other hand, NPC43 cells took shorter time to traverse through the pores and moved with higher speed along the trench sidewalls, which resulted in better guidance effect.

These results indicated that NPC43 cells showed different guidance effect and migration behavior after traversing through the porous membrane. Thus, the cell traversing probability, traversing time, and motility of NP460 and NPC43 cells after they passed through the pores were investigated systematically on the three-layer platforms.

### Lamellipodia morphology and cell motility of NP460 and NPC43 cells on three-layer platforms

To systematically investigate the effects of NP460 and NPC43 cell migration speed on the three-layer platforms, cells on the top and middle layers and cells traversed to the bottom layer were analyzed and shown in Figure 3A,B. When cells did not traverse to the bottom trenches, NP460 cell migration speed was higher than the NPC43 cells. Figure 3C shows that the large lamellipodia appeared around the NP460 cell body and some lamellipodia extended into the pores, while the NPC43 cell had many filopodia around the cell body and the filopodia attached to the sidewall of the pore. Lamellipodia and filopodia are actin filaments with different distributions in cells. Lamellipodia are the sheet-like protrusions around the NP460 cells that are related to mesenchymal migration. Filopodia are the needle-like protrusions around the NPC43 cells that sense the local environment and direct cell movement. Previous study showed that fewer large lamellipodial protrusions would slow down the cell migration [34]. Thus, NPC43 cells with more filopodia and fewer lamellipodial protrusions moved slower than NP460 cells on the top of the 3D platform. However, it was different when these two kinds of cells traversed to the bottom layer. Figure 3B shows that the traversed NP460 cells had a lower migration speed than the NPC43 cells. Once NPC43 cells migrated down to the trenches below, they moved at a higher speed. Figure 3D presents the more spread-out NP460 cell and the more round-shape NPC43 cell in the trench below. The movements of both cells are shown in Supplementary Video SV1. The traversing behavior of these two types of cells could be used to deduce how cells traverse through the endothelial layer *in vivo*. In the next section, lamellipodia morphology and cell shape will be used to explain the migration speed difference between these two cell lines.

### Lamellipodia morphology and cell shape on traversing behaviors of NP460 and NPC43 cells

After traversing through the porous membrane, all NP460 cells had elongated and spread-out morphologies as shown in Figure 3D. This is similar to the mesenchymal migration mode previously reported for the fibroblast cells [35,36]. Among the NPC43 cells traversed to the trenches below, 40% of the NPC43 cells alternated between the round shape shown in Figure 3D and the elongated shape during their migration in the trenches. A few small protrusions were also found on the traversed NPC43 cell in the trench as shown in Figure 3D [35–38]. These round-shape NPC43 cells could be related to the amoeboid migration mode with higher migration speed than the mesenchymal migration mode for the NP460 cells [35]. Amoeboid migration mode was reported for lymphoma, small-cell lung carcinoma, and small-cell prostate cancer [39–42]. Our results show that amoeboid migration mode could also apply to nasopharyngeal carcinoma cells in the tissue-like microenvironment with higher speed than immortalized nasopharyngeal epithelial cells.

**Figure 4:**
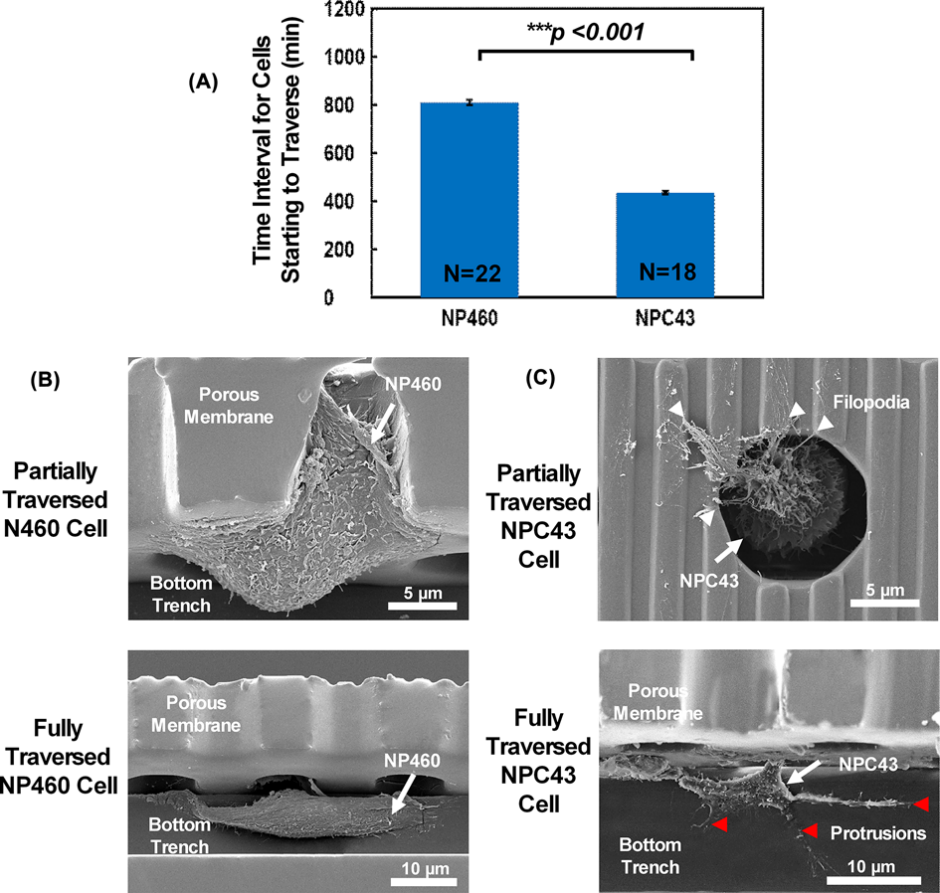
Cell traversing behaviour for NP460 and NPC43 cells Cell traversing behavior: (**A**) Time interval for NP460 and NPC43 cells starting to traverse through porous membrane after cell seeding. Cell morphologies of partially and fully traversed (**B**) NP460 and (**C**) NPC43 cells. White arrow indicated filopodia of NPC43 cell and red arrow represented protrusion of NPC43 cell.

The time interval between the cell seeding time and the time when the cells started to traverse through the porous membrane was analyzed as shown in Figure 4. NP460 cells took an average of 852 min to traverse through the pores compared with 422 min for NPC43 cells. Figure 4B,C show the cell shape of NP460 and NPC43 cells when they were partially and fully traversed to the trenches below the porous membrane. For partially traversed NPC43 cells, they often peeled off from the sidewalls when the samples were cut for cross-sectional imaging. Thus, a top view of a partially traversed NPC43 cell around a pore is shown in Figure 4C. The NP460 cells maintained the spread-out morphology with the sheet-like lamellipodia fully attached to the pore sidewalls when they were passing through the pores. They attached to the trench sidewalls after they were in the trenches and fully passed through the pores. Therefore, it could take longer time for the NP460 cells to fit into the 10 µm diameter pores in order to transverse through the pores and reach the trenches below. On the other hand, the NPC43 cells had round shape with smaller size than the pores and bundles of filopodia attached to the sidewall of the pores during traversing through the pores, and elongated shape with protrusions attached to the sidewall of the trenches after traversing. The NPC43 cells with the mutable shape and less spread-out lamellipodia took less time to traverse through the porous membrane to the trenches below. It could be inferred that the cancerous NPC43 cells are more reactive than the immortalized NP460 cells when traversing through porous membrane.

### Traversing behavior control of NP460 and NPC43 cells by guiding grating orientation and trench width

**Figure 5:**
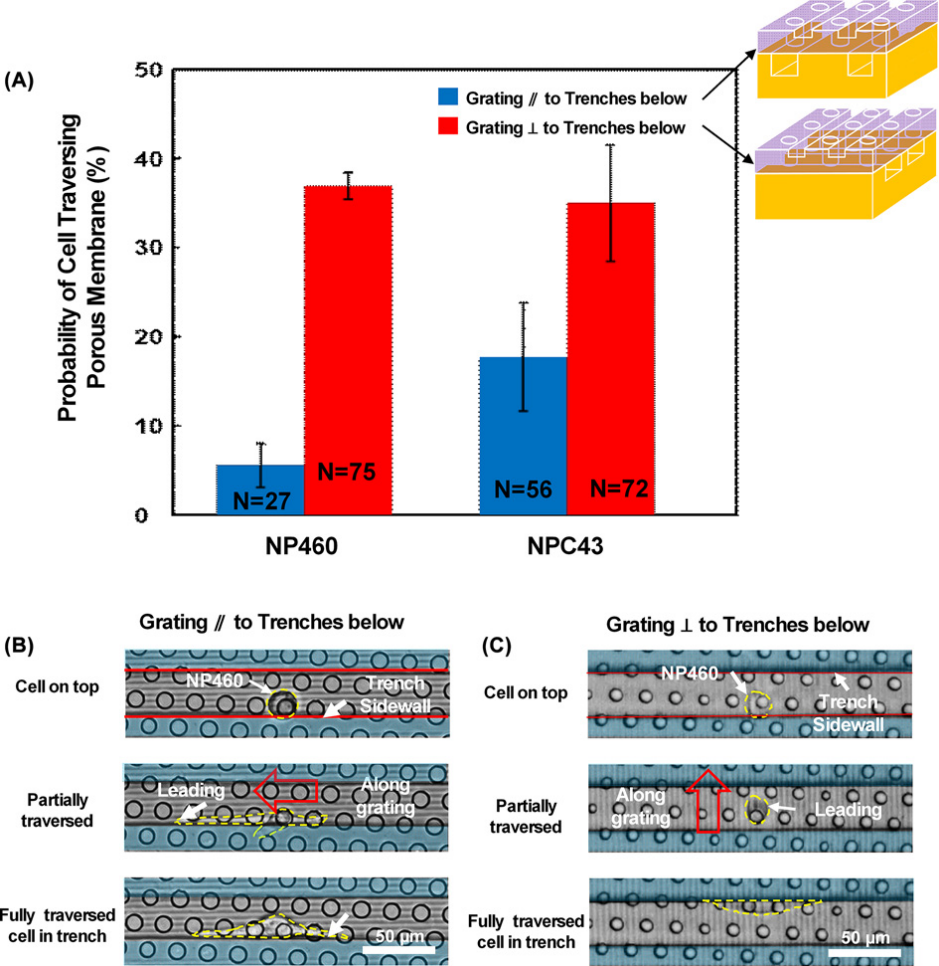
Cell traversing probability on platforms with different grating orientations (**A**) Control of NP460 and NPC43 cells traversing probability by top layer guiding grating orientation. NP460 cell traversing through porous membrane when guiding grating on top layer was (**B**) parallel and (**C**) perpendicular to trench below. Trench size was 30 μm in width and 15 μm in depth.

Guiding grating orientation and trench size were designed to control the cell traversing probability. Figure 5A shows the traversing probability of NP460 and NPC43 cells with the guiding grating orientation that were parallel and perpendicular to the trenches below, respectively. The trenches were 30 μm in width and 15 μm in depth. Traversing probability was defined as the percentage of the number of cells traversing through the porous membrane to the bottom trenches among the total number of cells on the platform. The results showed that the traversing probability on the platforms with the guiding grating perpendicular to the trenches below was larger than that with grating parallel to the trenches below for both NP460 and NPC43 cells. With guiding grating parallel to the trenches below, most of the cells traversed through the pores that were close to the trench sidewalls underneath as shown in Figure 5B since the sidewalls provided more contact area for the cells to attach. With guiding grating perpendicular to the trenches below, cells around the pores not next to the trench sidewall could still traverse to the trench below as shown in Figure 5C. The dimensions for the pores and the trenches were the same for Figure 5B,C. For Figure 5B, a scanning Nikon Eclipse Ni upright microscope with a 20× lens was used for the platforms with gratings parallel to the trenches below, and it was focused on the cells moving in the trenches of the bottom layer. For Figure 5C, in order to capture the cells traversing through the pores on the platforms with gratings perpendicular to the trenches below, a Leica TCS SP5 inverted microscope was used and it was focused on the top layer. The pores in both figures were 10 μm in diameter, and different microscopes were used for imaging on different layers to capture cells traversing the pores and in the trenches. The grating orientation perpendicular to the trenches below could shape the leading edge of the cell toward the trench sidewall for cell contact and to begin the traversing process. Thus, more NP460 and NPC43 cells could traverse through the porous membrane on the platforms with the guiding grating orientation perpendicular to the trenches below because of the higher probability for the cells to contact the trench sidewalls on the bottom layer.

**Figure 6:**
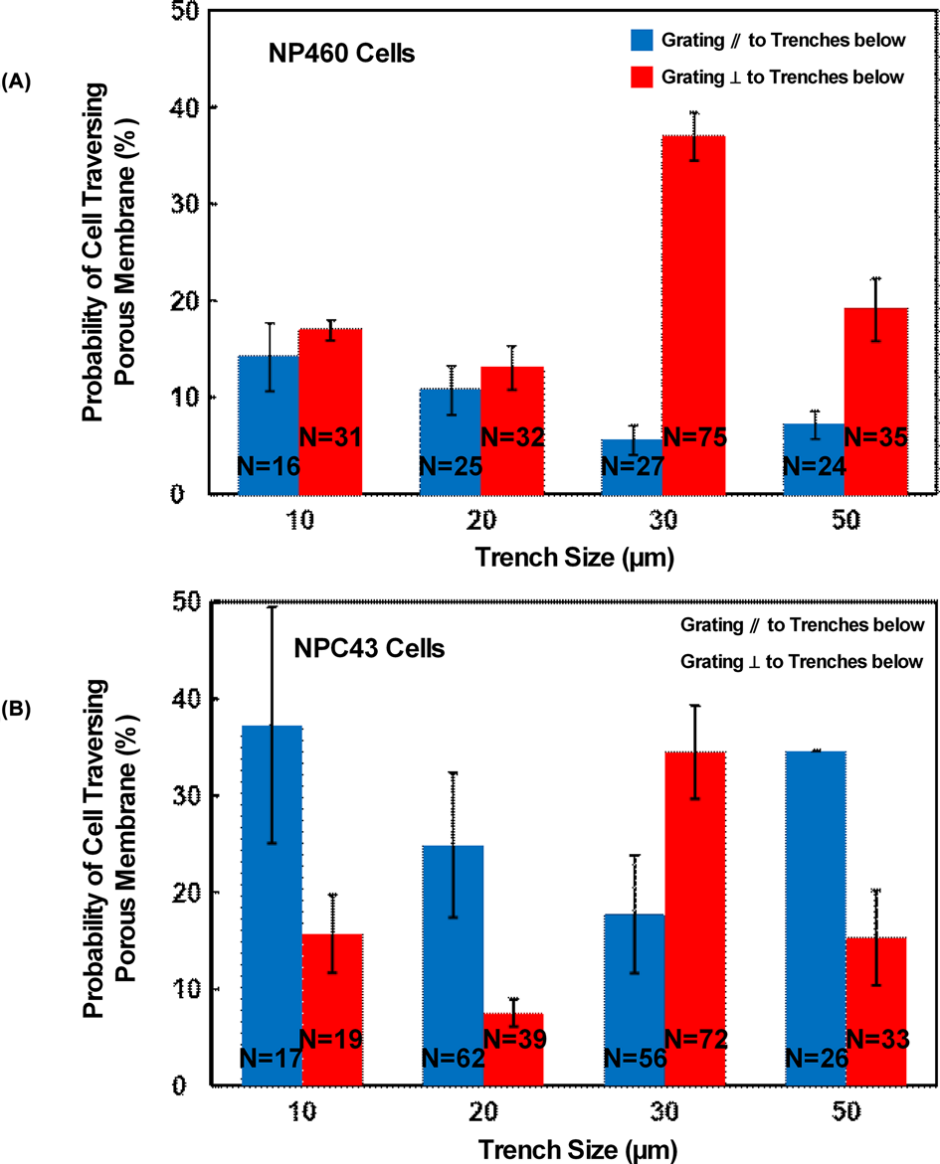
Cell traversing probability dependence on trench size Effect of trench size on bottom layer on (**A**) NP460 and (**B**) NPC43 cells traversing probability. Blue and red bars represent grating orientation parallel to and perpendicular to trenches below, respectively.

To further investigate other factors influencing traversing probability, the trench size was also adjusted because the distance to the trench sidewall and the pore density over the trenches could influence the cell sensing process and contact area. Figure 6 shows that 30 μm wide trenches with guiding grating orientation perpendicular to trenches below provided the highest probability for cells to traverse through the porous membrane for NP460 and NPC43 cells. The lowest probability of NP460 cells to traverse through the porous membrane was for the 30 μm wide trenches with guiding grating orientation parallel to the trenches below as shown in Figure 6A. When the grating orientation on the top layer was perpendicular to the trenches below, the leading edges of the cells were directed to contact the sidewalls of the trenches on the bottom layer, which resulted in higher probability for cells to migrate to the bottom trenches. For narrower trenches with 10 or 20 μm width in the bottom layer, the trench width was smaller or close to the cell size. Cells would be confined in a tight space, which could reduce the traversing probability. For larger trench width of 50 µm, cells had to reach a longer distance for the trench sidewalls, which could also reduce the traversing probability compared to the 30 µm wide trenches.

Figure 6B shows higher NPC43 cell traversing probability on the platforms with guiding grating perpendicular to the 30 µm wide trenches in the bottom layer. Since filopodia along the leading edges of the NPC43 cells would point toward the trench sidewalls, cells were guided to contact the trench sidewalls that helped the cells to traverse toward the trenches. However, traversing probability of NPC43 cells on platforms with grating parallel to the trenches below was higher than that the ones with grating perpendicular to trenches that were 10, 20, and 50 μm wide in the bottom layer as shown in Figure 6B. This might be related to the many filopodia extended from the NPC43 cells, which were different from the sheet-like lamellipodia extended from the NP460 cells. NPC43 cells typically elongated along the direction of the guiding grating, with the filopodia extended from the cell bodies. When the trench width was 10 or 20 µm, only one row of pores would be exposed to the trench below. The elongated NPC43 cells on platforms with guiding grating parallel to the trenches below had a higher chance of contacting the pores compared to cells elongated with a 90° offset when the grating was perpendicular to the trenches below. Therefore, traversing probability of NPC43 cells was higher on platforms with grating parallel to the 10 and 20 µm wide trenches. On the other hand, for 30 µm wide trenches, two rows of pores would be exposed to the trench below, providing more opportunities for the NPC43 cells to reach the trench sidewalls and the cell elongation orientation was not important. For the 50 µm wide trenches, at least one row of pores will be away from the trench sidewalls. Under this condition, having cells elongated along the grating that was parallel to the trenches below resulted in a higher chance of cells contacting the pores, and hence a higher traversing probability compared with platforms with grating that was perpendicular to the trenches.

By designing the 3D platform dimensions and layouts, such as the guiding grating orientation, trench width, layer thickness, and pore size, different types of cells could be controlled either to stay on top of the porous membrane or to traverse to the trenches below. This will be useful to understand the cell invasiveness through porous membrane, and perhaps could lead to designs that could keep certain cells from migrating through epithelial layers. In addition to the physical cues from the 3D platforms, the migration behaviors of the NP460 and NPC43 cells are closely related to lamellipodia and filopodia protrusions from the cells, which are associated with phosphoinositide 3-kinases (PI3K) that regulate signaling pathway [43,44]. Thus, it will be useful to study the effect of molecular signal transduction of PI3K inhibitor pathway.

## Conclusion

In the present study, 3D platforms with precisely controlled dimensions were designed and fabricated with multiple layers to mimic tissue microenvironment with ECM topography, porous interface, and trenches below. Cell migration behaviors of immortalized nasopharyngeal epithelial NP460 cells and carcinoma NPC43 cells on the transparent 3D platforms were monitored over time. NP460 and NPC43 cells were guided along the grating orientation but they lost the direction preference both on the porous substrates and the two-layer platforms without trenches below. On the three-layer platforms, NP460 cells showed no direction preference but NPC43 cells migrated along the trench orientation on the bottom layer. Cell migration speed for NP460 cells on the top layer was higher than NPC43 cells. But the traversed NP460 cells had lower migration speed compared to the NPC43 cells.

Lamellipodia spreading and cell shape were investigated, and they were related to the cell motility and traversing activity. When NPC43 cells were on the top and middle layers without traversing to the trenches below, they had fewer lamellipodia protrusions and moved at lower migration speed compared to NP460 cells. However, the mutable cell shape of NPC43 cells after traversing to the trenches below resulted in the combined amoeboid migration and mesenchymal migration modes, which led to higher migration speed after traversing to the trenches below compared with the mainly mesenchymal migration mode of NP460 cells. The cell traversing probability through porous membrane was controlled by the guiding grating orientation and trench size as they were related to the cell contact area, distance to the trench sidewalls, and the confinement inside the trenches. For 30 µm wide trenches in the bottom layer, higher cell traversing probability was found on the platforms with guiding grating perpendicular to the trenches as the extended lamellipodia or filopodia along the leading edges of the cells would point towards the sidewalls. Cells were guided to reach through the pores and contact the trench sidewalls on the bottom layer, which helped the cells to traverse towards the trenches. On the other hand, when the guiding grating was parallel to the trenches, the protrusions from the leading edges of the cells would point along the trench orientation and not toward the trench sidewalls, making it more difficult to contact the trenches, hence less cells were able to traverse to the trenches. Cell traversing probability on platforms with 30 μm wide trenches below was the highest when guiding grating was perpendicular to trenches below and the lowest when guiding grating was parallel to trenches below.

The developed 3D platforms provided a microenvironment that mimic a tissue including an extracellular matrix with topography and a blood vessel with porous interface. The dimensions, designs, and surface properties of the platforms could be controlled precisely, hence these biomimetic microsystems could be used to study the migration behaviors of the immortalized nasopharyngeal epithelia cells and carcinoma cells. With the transparent PDMS platforms, cell migration through the porous membrane could be monitored to distinguish migration behaviors of cancer and normal cells. By understanding how these cells move differently on the designed platforms, it could lead to the ability to identify the metastatic potential of cancer cells. In the present study, it was mainly focused on the physical cues of the 3D platforms such as contact areas and cell confinement influences on cell migration. Further study will be extended to applying various disease-related molecules on the 3D platforms to gain the knowledge of biological effects in additional to the physical microenvironment on cancer metastasis.

## Supplementary Material

Supplementary Figure S1

Supplementary Video SV1

## Abbreviations

EBV: Epstein–Barr virus
ECM: extracellular matrix
NPC: nasopharyngeal carcinoma
