# Supplementary material for: Migration of immortalized nasopharyngeal epithelia and carcinoma cells through porous membrane in 3D platforms
Source: Biosci Rep. 2020 Jun 4;40(6):BSR20194113. doi: 10.1042/BSR20194113 (PMC7273909; doi:10.1042/BSR20194113)
Supplement: Supplementary Figure S1 [file BSR-2019-4113_supp.pptx]

## Slide 1
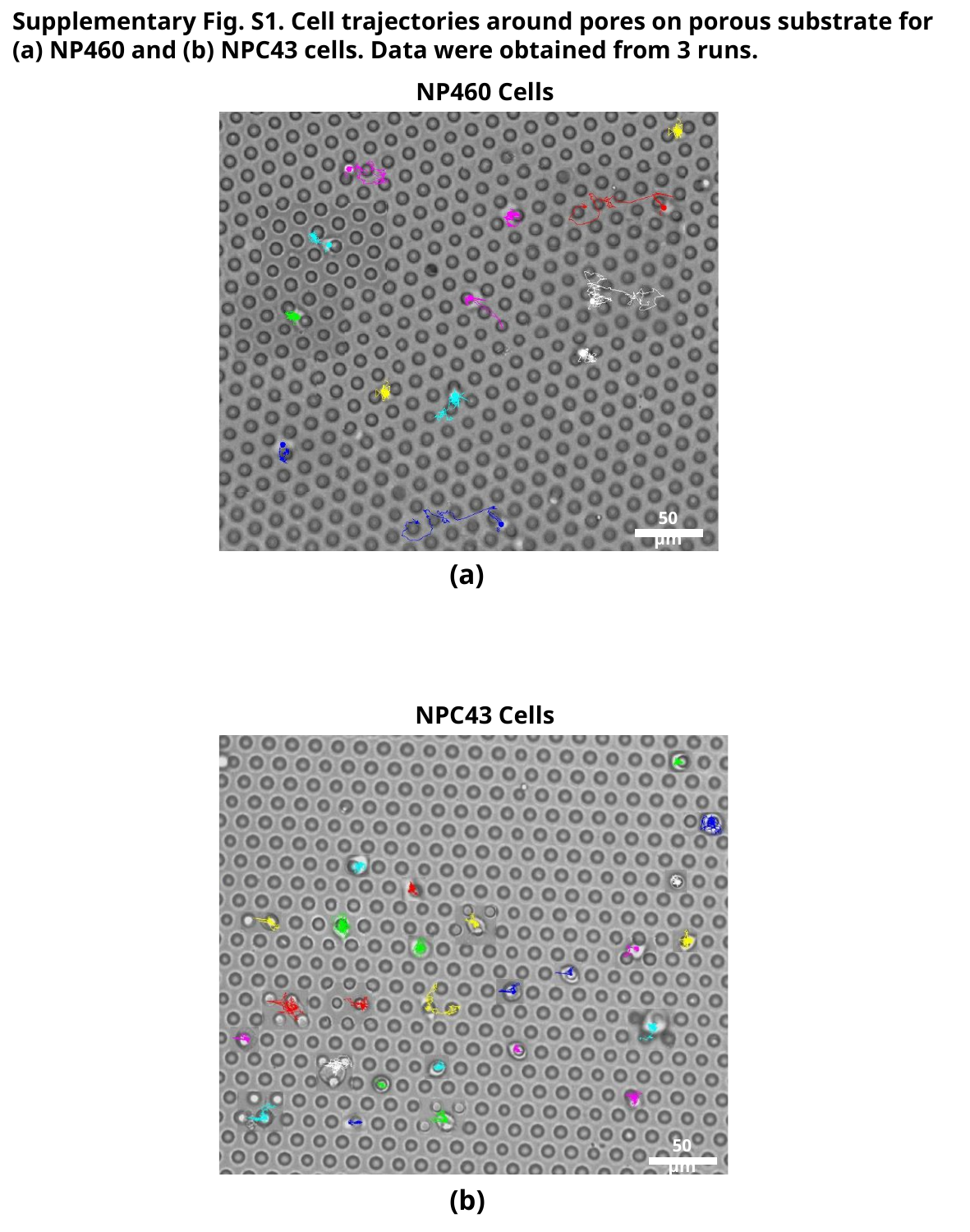

Supplementary Fig. S1. Cell trajectories around pores on porous substrate for (a) NP460 and (b) NPC43 cells. Data were obtained from 3 runs.
NP460 Cells
(a)
50 μm
NPC43 Cells
50 μm
(b)
