# Supplementary material for: Migration of immortalized nasopharyngeal epithelia and carcinoma cells through porous membrane in 3D platforms
Source: Biosci Rep. 2020 Jun 4;40(6):BSR20194113. doi: 10.1042/BSR20194113 (PMC7273909; doi:10.1042/BSR20194113)
Supplement: Supplementary Video SV1 [file BSR-2019-4113_supp1.pptx]

## Slide 1
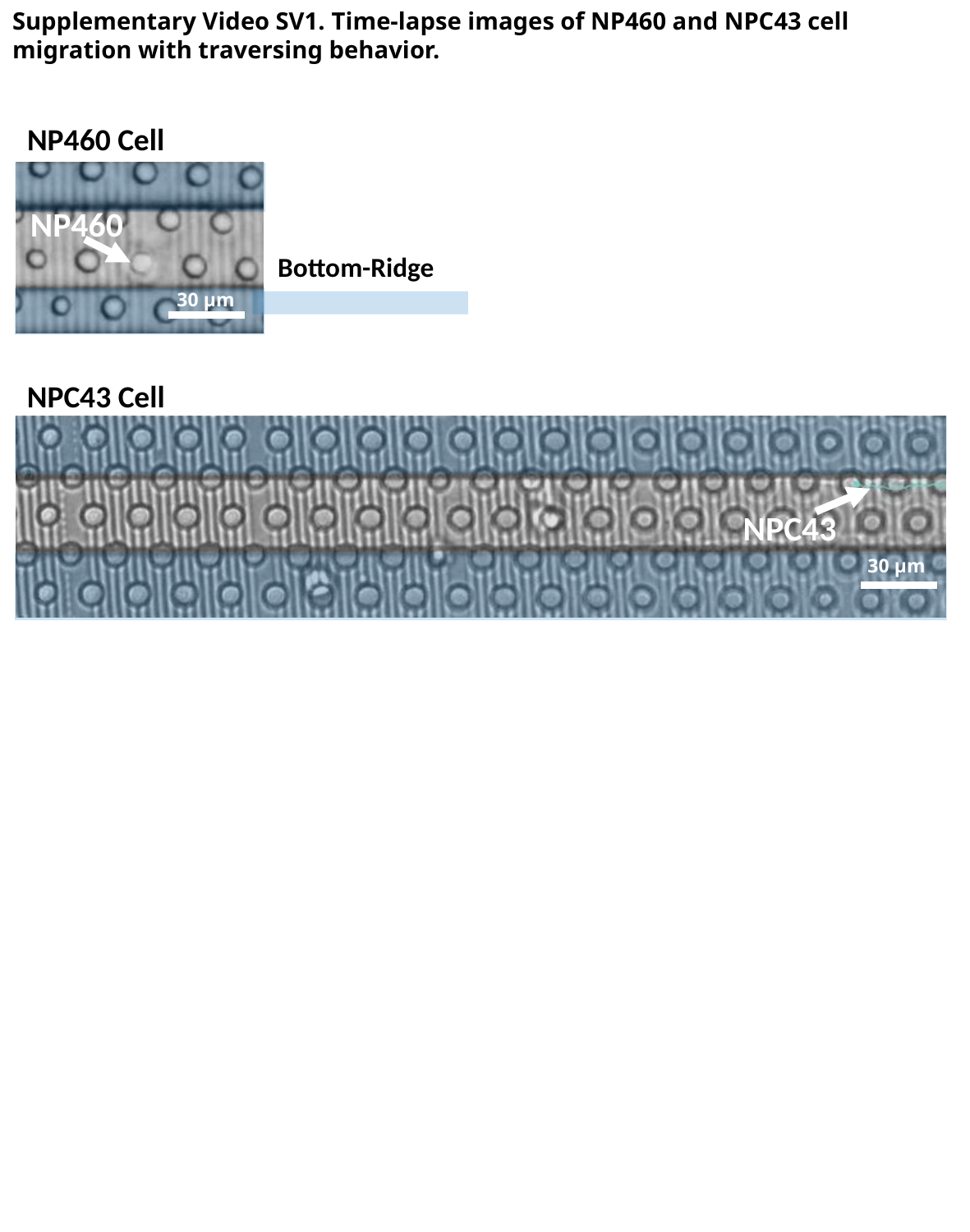

Supplementary Video SV1. Time-lapse images of NP460 and NPC43 cell migration with traversing behavior.
NP460 Cell
NP460
Bottom-Ridge
30 μm
NPC43 Cell
NPC43
30 μm
